# Hysteria with Probable Gastric Ulcer

**Published:** 1892-10

**Authors:** Allen A. Jones

**Affiliations:** Lecturer on, and Instructor in, Practice, Medical Department, University of Buffalo


### HYSTERIA WITH PROBABLE GASTRIC ULCER.

BY ALLEN A. JONES, M. D.,
Lecturer on, and Instructor in, Practice, Medical Department, University of Buffalo.

THE chief points of interest in this case are hysterical cough, trance, convulsions, fainting spells, esophageal stricture, paralysis of right leg, associated with profuse hematemesis, persistent vomiting, regurgitation and nervous belching.

A young woman, E. P., aged 20, presented herself May 16, 1892, and gave the following history: Father died of heart disease at thirty-two. Mother living and well. Never had a brother. One sister living and healthy; one died at sixteen; cause of death unknown. Patient was a healthy infant and child until her tenth year, when she menstruated first. From that date she suffered much from intercostal neuralgia. Between her tenth and fifteenth years she had a dry, hacking cough, lasting one year. When about thirteen, she had a trance, commencing at 7.30 A. M. on May 12, 1885, and continuing until 3.30 P. M. the day following. The same month she had convulsions, lasting, one after another, eighteen hours a day, and these were frequent and severe until September. During that period stricture of the esophagus was so persistent that gavage was practised several months. Late in the Autumn of that year she grew better, but had not the use of her right leg, so could not walk for some time. Since that time she has been very weak and tormented by epigastric and ovarian pain. She has had leucorrhea since she was thirteen years old. Hematemesis occurred first at the time of her trance, and since that event she has vomited blood, sometimes three or four times a day, two or three days in a week, sometimes with intermissions of a month or six weeks. She spent the Summer of 1890 on Lake Erie shore, but vomited nearly everything she ate, and, upon returning to Buffalo, fainted three or four times a day, vomited frequently a teacupful of blood, and became fearfully blanched. Last Summer, was better than usual until September, when she again commenced vomiting food and blood; had severe epigastric pain, grew steadily paler and weaker, was constipated, but occasionally had diarrhea with bloody stools.

May 16, 1892.—At present she regurgitates her food, vomits blood frequently, has pain, made worse by eating. gastric and intestinal flatulency with sour stomach. She is constipated, has a capricious appetite, alternating with total anorexia; craves sour things. She frequently has "fainting spells" of five or ten minutes' duration. Sleeps poorly ; no somnambulism. Vision apparently normal, its field not contracted, no nystagmus, no color-blindness. Last menstruated four months ago, flow was scanty. Leucorrhea is profuse. Uterus otherwise normal.

Blood by D'Henocque's hematospectroscope registers by the plate forty, by the spectroscope forty-five. Obviously the hemoglobin is much reduced. About two inches above the coccyx is a very tender spot, but nothing of the kind in the other spinal regions. Reflexes, normal. No areas of anesthesia or paresthesia. No conjunctival anesthesia. Lungs, normal. Over precordium is heard a soft, blowing, systolic, apical murmur. Pulse, fairly strong, regular, with moderate arterial tension. Liver and spleen, normal. Epigastric tenderness located a little to right of median line. Stomach, normal size ; no clapotage. Sensitiveness over ovaries. Examination of abdomen otherwise negative. Urine, phosphatic.

